# Targeted Restoration of T-Cell Subsets by a Fluorinated Piperazine Derivative β-Cyclodextrin Complex in Experimental Pulmonary Inflammation

**DOI:** 10.3390/molecules30132741

**Published:** 2025-06-25

**Authors:** Valentina Yu, Marina Balabekova, Assel Ten, Tolganay Zharkynbek, Sulev Koks, Milana Alimova, Raushan Koizhaiganova, Meruyert Mussilim, Aigul Malmakova, Tulegen Seilkhanov, Khaidar Tassibekov

**Affiliations:** 1 Laboratory of Synthetic and Natural Medicinal Compounds Chemistry, A.B. Bekturov Institute of Chemical Sciences, 106 Sh. Ualikhanov St., Almaty 050010, Kazakhstan; yuvkconst@gmail.com (V.Y.); assel.ten@gmail.com (A.T.); k_raushan@yahoo.com (R.K.); malmakova@mail.ru (A.M.); kh.tassibekov@ihn.kz (K.T.); 2 Pathological Physiology Department, Asfendiyarov Kazakh National Medical University, 94 Tole-bi St., Almaty 050000, Kazakhstan; alimova.milana1999@gmail.com (M.A.); muslim.m@kaznmu.kz (M.M.); 3 Health Futures Institute, Murdoch University, 90 South St., Perth, WA 6150, Australia; sulev.koks@murdoch.edu.au; 4 Laboratory of Engineering Profile NMR Spectroscopy, Sh. Ualikhanov Kokshetau State University, 76 Abai St., Kokshetau 020000, Kazakhstan; tseilkhanov@mail.ru

**Keywords:** complex of dimethyl[(4-benzhydrylpiperazin-1-yl)(2-fluorophenyl)methyl]phosphonate with β-Cyclodextrin ((***o*-Fph**)**PPhβCD**), Polyoxidonium (PO), acute pneumonia, regulatory T cells, immunomodulation

## Abstract

Acute pneumonia is frequently accompanied by immune suppression, particularly affecting T-cell subsets, such as CD4^+^, CD4^+^CD25^+^, and CD4^+^CD25^+^FoxP3^+^, which are critical for immune regulation. This study evaluates the immunomodulatory potential of a novel fluorinated piperazine-based aminophosphonate, complexed with β-cyclodextrin ((***o*-Fph**)**PPhβCD**), comparing it with the clinically approved agent Polyoxidonium (PO) in a rat model of oleic acid-induced acute pneumonia. Flow cytometric analysis revealed that (***o*-Fph**)**PPhβCD** significantly restored CD4^+^ and CD4^+^CD25^+^ T-cell levels and induced a sustained reduction in regulatory CD4^+^CD25^+^FoxP3^+^ cells, suggesting enhanced effector immune activity. While PO provided early immunorestorative effects, (***o*-Fph**)**PPhβCD** exerted a more prolonged response, which was particularly evident by day 14. Structural confirmation of the inclusion complex was achieved through IR and NMR spectroscopy. These findings highlight (***o*-Fph**)**PPhβCD** as a promising immunotherapeutic candidate that is capable of rebalancing immune cell populations and supporting host defense mechanisms during acute pulmonary inflammation.

## 1. Introduction

Pneumonia remains one of the leading causes of morbidity and mortality worldwide, according to the World Health Organization (WHO). The disease disproportionately affects vulnerable populations, including children under five years of age [1] and adults over seventy [2]. Annually, approximately 450 million people worldwide suffer from pneumonia, with the severity and outcomes of the disease strongly influenced by patient age and the presence of chronic comorbidities [3,4,5]. As the most severe form of acute lower respiratory tract infection, pneumonia is characterized by alveolar infiltration with purulent exudate due to microbial infection and remains a significant contributor to global mortality [6]. The increasing prevalence of antimicrobial resistance, combined with the limited effectiveness of conventional antibiotics in immunocompromised patients, underscores the urgent need to explore novel therapeutic approaches, particularly those targeting immune regulation.

T lymphocytes are critical regulators of host defense during pulmonary infections. CD4^+^ T-helper cells (Th1, Th2, Th17) orchestrate the immune response by activating macrophages, neutrophils, and B lymphocytes, while CD8^+^ cytotoxic T cells eliminate pathogen-infected cells. However, the hyperactivation of these subsets can drive excessive inflammation and cause collateral tissue damage. In both acute and chronic inflammatory settings, the local immune microenvironment plays a pivotal role in influencing T cell differentiation, maturation, and effector functions [7]. In the specific context of pneumonia, CD4^+^ regulatory T cells (Tregs), characterized by the expression of CD25^+^ and FoxP3^+^, serve a central function in suppressing pathological immune activation and promoting tissue repair [8,9,10]. Dysregulation of the Treg compartment can result in either excessive inflammation or impaired pathogen clearance, both of which are detrimental to patient outcomes.

Modern immunomodulatory therapies aim to restore immune balance by enhancing regulatory mechanisms and limiting hyperinflammation. These therapies can modulate cytokine profiles, improve Treg function, and reduce the risk of progression to fibrosis or chronic inflammation. Importantly, they can also synergize with antibiotics and mitigate the complications associated with antibiotic overuse. Consequently, the search for novel immunomodulatory agents with well-defined cellular mechanisms is of critical importance for the effective treatment of inflammatory lung diseases, including acute pneumonia.

Cyclodextrins (CDs) and, in particular, β-cyclodextrin (β-CD) are widely used in pharmaceutical research, due to their ability to form non-covalent inclusion complexes with hydrophobic drug molecules. These inclusion complexes often improve the aqueous solubility, chemical stability, and bioavailability of the active compound, while offering the potential for controlled or sustained release. Such properties make CDs attractive carriers for poorly soluble drugs and allow for the modulation of pharmacokinetic profiles without altering the compound’s pharmacodynamic mechanism. In this context, complexation with β-CD may enhance in vivo exposure, thereby enabling pharmacological effects that are otherwise limited by poor solubility or overly rapid metabolism.

**Figure 1:**
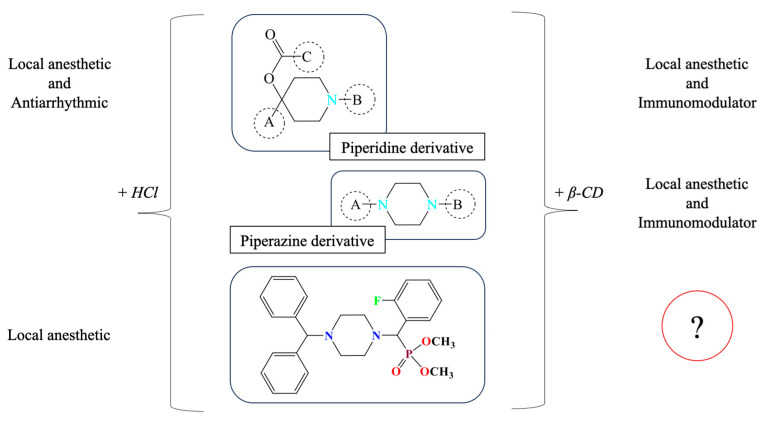
Background rationale for selecting (***o*-Fph**)**PPhβCD** as a lead compound for immunological evaluation. Inclusion complexation with β-cyclodextrin is known to enhance solubility and bioavailability, which may indirectly influence the observable pharmacological profile in vivo. However, the intrinsic pharmacodynamic activity of the active compound remains structurally driven.

The present study focuses on synthetically derived azaheterocycles as a new class of candidate immunomodulators. The selection of the target compound was based on a series of previously published experimental results, as summarized in Figure 1, which collectively suggest promising pharmacological potential for these agents.

Previous research has demonstrated that the fluorobenzoate derivatives of ethynylpiperidols in hydrochloride form exhibit local anesthetic and antiarrhythmic activity [11]. Interestingly, similar compounds complexed with β-CD, such as piperidine derivatives containing a (naphthalen-1-yloxy)but-2-yn-1-yl moiety, have also shown pronounced biological effects [12]. In particular, the β-CD complex of the propionate ester of ethynylpiperidol was associated with the detection of immunomodulatory activity, as evidenced by reduced inflammation in models of lead and cadmium intoxication [13]. This suggests that β-CD inclusion may play a key role in enhancing systemic availability and prolonging the pharmacological effect.

Building on these insights, our group explored certain derivatives of piperazine—a saturated six-membered nitrogen-containing heterocycle—formulated as β-CD inclusion complexes. These compounds displayed both local anesthetic [14] and immunoregulatory properties [15]. More recently, we synthesized a set of aminophosphonates derived from benzhydrylpiperazine, incorporating *ortho*-, *meta*-, and *para*-fluorinated phenyl substituents [16]. Their hydrochloride salts exhibited local anesthetic efficacy in infiltration models [17]. Among these, the β-CD complex of dimethyl[(4-benzhydrylpiperazin-1-yl)(2-fluorophenyl)methyl]phosphonate, denoted as (***o*-Fph**)**PPhβCD**, was selected for further investigation due to its superior solubility and formulation stability compared to the meta- and para-isomers. This study was designed to evaluate the complex’s immunomodulatory potential in a rat model of acute pneumonia, with a particular focus on CD4^+^ T-cell subpopulations, including Tregs. For benchmarking, Polyoxidonium, a clinically approved immunomodulator, was used as a reference.

## 2. Results and Discussion

Certain nitrogen-based heterocyclic derivatives have been reported to exhibit immunomodulatory activity, targeting various endogenous and exogenous pathways and influencing the parameters of immune regulation in a compound-specific manner. These agents possess the ability to influence the magnitude, nature, duration, and efficacy of immune reactions, thereby shaping the intricate cascade of host defense mechanisms [18]. Immunomodulators are designed to optimize immune responses, either by enhancing host resistance against infections or by attenuating any excessive immune activation that may result in tissue damage. A promising strategy for the treatment of aseptic pneumonia involves modulation of the immune microenvironment. To achieve more effective immune regulation and tissue regeneration, novel anti-inflammatory and immunomodulatory agents may improve the immunological milieu and promote cellular homing and pulmonary tissue repair [19].

**Scheme 1:**
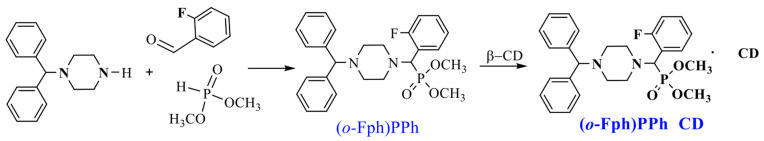
Synthesis of dimethyl[(4-benzhydrylpiperazin-1-yl)(2-fluorophenyl)methyl]phosphonate ((*o*-Fph)PPh) [16] and its complex with β-cyclodextrin ((***o*-Fph**)**PPhβCD**).

The target aminophosphonate was synthesized via a “one-pot” Kabachnik–Fields three-component reaction involving 1-benzhydrylpiperazine, *o*-fluorobenzaldehyde, and dimethyl phosphite [16] (Scheme 1).

At the initial stage of the study, β-CD inclusion complexes (ICs) were synthesized for all three fluorinated isomers—the *ortho*-, *meta*-, and *para*-isomers of dimethyl[(4-benzhydrylpiperazin-1-yl)(fluorophenyl)methyl]phosphonate. However, the ICs of the *meta*- and *para*-isomers exhibited poor water solubility and physical instability. At concentrations that permitted dissolution, these complexes showed no significant activity in the preliminary bioassays. Furthermore, mild heating of their solutions led to visible degradation (e.g., yellowing), and cooling often resulted in rapid precipitation and local tissue irritation upon injection. Due to these limitations, only the *ortho*-isomer IC ((***o*-Fph**)**PPhβCD**) was selected for further biological evaluation.

The IC (***o*-Fph**)**PPhβCD** was synthesized by mixing an ethanol solution of the phosphonate ((*o*-Fph)PPh) with an aqueous solution of β-CD in a 1:1 molar ratio. The resulting mixture was subjected to ultrasonic treatment for 30 min at 50 °C and subsequently placed in a drying oven at 50–55 °C to allow solvent evaporation. This procedure yielded the target inclusion complex as a white powder, with an efficiency of 96.3%.

The formation of (***o*-Fph**)**PPhβCD** was confirmed by infrared (IR) and nuclear magnetic resonance (NMR) spectroscopy. Figures S1–S5 in the Supplementary Materials display the IR spectra of β-CD, the free phosphonate ((*o*-Fph)PPh), and the resulting complex (***o*-Fph**)**PPhβCD**. A comparative analysis of the characteristic absorption bands of specific functional groups was conducted (Table 1).

Despite the apparent similarity between the IR spectra of (***o*-Fph**)**PPhβCD** (Figure S2) and that of β-CD (Figure S1), as shown in the overlay (Figure S4), notable spectral shifts, including displacement of the P=O, C=N, and C–F absorption bands, reduced intensity of P–C bond absorption, and alterations in the spectral profile within the aromatic region (1585–1699 cm^−1^) provide strong evidence for the formation of the intended inclusion complex.

Furthermore, the structures of both the IC (***o*-Fph**)**PPhβCD** complex (Figure 2) and the guest molecule (*o*-Fph)PPh were confirmed by ^1^H and ^13^C nuclear magnetic resonance (NMR) spectroscopy (Figures S6–S12).

In the ^1^H NMR spectrum of (*o*-Fph)PPh (Figure S6), a singlet at δ 2.34 and a multiplet at δ 2.52–2.80 were assigned to the piperazine protons H-2ax,6ax,2eq,6eq and H-3ax,5ax,3eq,5eq, respectively. The methoxy protons H-17,17,17 and H-31,31,31 appeared as two multiplet signals at δ 3.37–3.40 and δ 3.75–3.78, respectively. The methine protons H-14 and H-7 were observed as multiplets at δ 4.37–4.40 and δ 4.43–4.47, respectively. The aromatic protons H-26,28; H-9–13,29,19–23; and H-27 of (o-Fph)PPh appeared as multiplets in the regions δ 7.11–7.15, δ 7.21–7.75, and δ 7.81–7.92, respectively.

In the ^1^H NMR spectrum of the (***o*-Fph**)**PPhβCD** (Figure S9), the piperazine protons H-2ax, 6ax, 2eq, 6eq and H-3ax,5ax,3eq, 5eq of the guest molecule ((*o*-Fph)PPh) appeared as two multiplets in the ranges of 2.27–2.45 ppm and 2.48–2.77 ppm, respectively. The methoxy protons H-17,17,17 and H-31,31,31 were detected as two sets of multiplet signals at 3.58–3.61 ppm and 3.76–3.77 ppm. A singlet at 4.19 ppm and a multiplet at 4.34–4.38 ppm were attributed to the methine protons H-14 and H-7, respectively. The aromatic region displayed multiplet signals at 7.08–7.12, 7.18–7.55, and 7.68–7.71 ppm, corresponding to the aromatic protons H-26,28; H-9–13,29,19–23; and H-27 of the “guest” compound.

For the “host” molecule (β-CD), the oligosaccharide protons H-2 and H-4 were observed as multiplets at 3.25–3.28 ppm and 3.30–3.33 ppm, respectively. Additional multiplet signals for protons H-5, H-3, and H-6 appeared at 3.51–3.53, 3.56–3.58, and 3.60–3.63 ppm. A singlet at 4.79 ppm was assigned to the anomeric proton H-1 of the oligosaccharide ring.

The ^13^C NMR spectrum of (*o*-Fph)PPh (Figure S7) was characterized by signals corresponding to the carbon atoms of the piperazine fragment at δ 52.23 (C-2,6), 52.91, and 54.70 (C-3,5). Methoxy carbon atoms C-17 and C-31 were observed at δ 56.40 and 56.89, respectively. The methine carbon atoms of the (*o*-Fph)PPh molecule appeared at δ 58.25 (C-14) and δ 75.36 (C-7). Aromatic carbon atoms were registered at δ 115.92 and 117.33 (C-24,26), 124.82 (C-28), 127.54 (C-11,21), 127.64 (C-10,12,22,20), 129.09 (C-9,13,23,19), 130.97 (C-29), 132.07 (C-27), 142.19 (C-8,18), and 160.03 and 162.46 (C-25).

The signals corresponding to the oligosaccharide carbon atoms of β-CD were observed at 60.63 ppm (C-6), 72.46 ppm (C-5), 73.75 ppm (C-2), 73.75 ppm (C-3), 82.22 ppm (C-4), and 102.70 ppm (C-1).

Assignment of the proton and carbon resonances was supported by two-dimensional NMR experiments, including COSY (Figures S8 and S11) and HMBC (Figure S12) spectra of the (***o*-Fph**)**PPhβCD**.

It was found that the chemical shift changes in the ^1^H NMR spectrum of β-CD (Table 2) primarily affected the protons that were oriented toward the inner cavity of cyclodextrin: H-3 (Δδ = 0.05 ppm) and H-5 (Δδ = 0.03 ppm). For the protons located on the exterior of the cyclodextrin cavity, the observed changes were within Δδ = 0.01–0.04 ppm for H-1 and H-4, and Δδ = 0.06 ppm for H-6.

These results indicate the formation of mixed inclusion complexes of (***o*-Fph**)**PPhβCD**. During the supramolecular self-assembly of the guest molecules with the cyclodextrin oligomer, notable changes in the chemical shift values of the aromatic protons H-26, H-27, and H-28 of (o-Fph)PPh (Δδ = 0.03–0.17 ppm) were observed, suggesting that these protons are probably involved in interactions with the glucopyranose protons of β-CD.

To explore the biological implications of the synthesized compound, we applied the characterized (***o*-Fph**)**PPhβCD** to a well-established murine model of acute pneumonia, which mirrors critical immunopathological features of the human condition.

**Figure 3:**
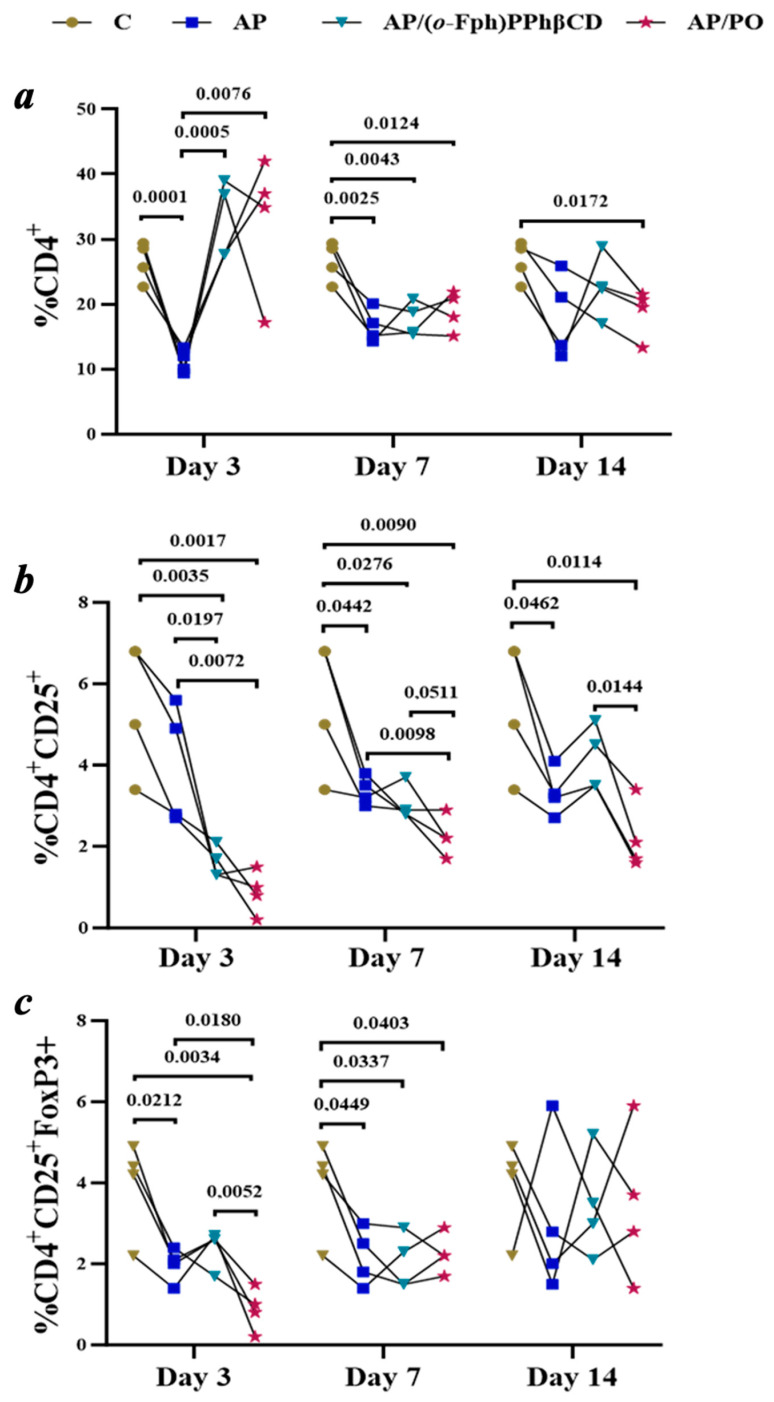
Dynamics of T-Lymphocyte subpopulation changes (CD4^+^, CD4^+^CD25^+^, CD4^+^CD25^+^FoxP3^+^) in the spleens of rats with acute pneumonia on days 3, 7, and 14 across the following groups: Control (C), untreated pneumonia (AP), treatment with (***o*-Fph**)**PPhβCD** (AP/(***o*-Fph**)**PPhβCD**), and treatment with Polyoxidonium (AP/PO). Panels: (**a**)—CD4^+^ T cells (%), (**b**)—CD4^+^CD25^+^ cells (%), (**c**)—CD4^+^CD25^+^FoxP3^+^ regulatory T cells (%). Results are presented as mean values ± standard deviation. Statistical significance was calculated using Student’s *t*-test. The numeric values shown in the figure indicate the exact *p*-values for statistically significant differences compared to the respective comparison group.

*Characterization of the AP group (acute pneumonia).* The AP group consisted of animals with experimentally induced acute pneumonia that did not receive any specific therapeutic intervention. This model recapitulates key pathophysiological features of bacterial pneumonia, including pronounced inflammatory responses, immune dysregulation, and suppression of the effector arm of the immune system. The dynamic profile of immunological parameters is illustrated in Figure 3.

The results of flow cytometric analysis are presented in Figure 3, illustrating the temporal dynamics of key CD4^+^ T-cell subpopulations in spleen samples across all experimental groups. The data provide insight into how acute pneumonia and the subsequent treatments influence effector and regulatory immune cell compartments over time.

***a***: Changes in CD4^+^ T-lymphocyte levels are apparent in control animals (C), untreated animals with acute pneumonia (AP), and those receiving treatment with (**o-Fph**)**PPhβCD** (AP/(**o-Fph**)**PPhβCD**) or Polyoxidonium (AP/PO) on days 3, 7, and 14. A pronounced decline in CD4^+^ levels was observed in the pneumonia group, with partial recovery under both treatment regimens.

***b***: The dynamics of CD4^+^CD25^+^ T cells reflect alterations in the regulatory arm of the immune response. A decrease in this population was noted in the AP group, particularly on day 14. Treatment with (**o-Fph**)**PPhβCD** facilitated recovery in these cells, while PO exerted a less pronounced effect on this parameter.

***c***: A reduction in the levels of CD4^+^CD25^+^FoxP3^+^ regulatory T cells in acute pneumonia suggests a disruption in immune tolerance mechanisms. Both (**o-Fph**)**PPhβCD** and PO further suppressed Treg levels, potentially indicating an enhancement of effector immune responses.).

*Early stage of acute pneumonia* (*day 3*). By day 3, a marked decline in the level of CD4^+^ T-lymphocytes was observed (M = 1.2, SD = 1.8), representing a greater than 2.3-fold reduction compared to the control group (M = 26.6, SD = 3.0; *p* < 0.0001). This finding indicates the transient suppression of cell-mediated immunity and disruption in the coordination of the immune response, a phenomenon commonly seen during the early phase of disease progression that is influenced by multiple comorbid factors. The level of CD4^+^CD25^+^ activated T cells was also reduced compared to the control group (M = 4.0, SD = 1.5 vs. M = 5.5, SD = 1.6), although the difference did not reach statistical significance (*p* = 0.2117). This may reflect the insufficient activation of effector immune mechanisms.

*Intermediate stage of acute pneumonia* (*day 7*). By day 7, the partial recovery of CD4^+^ T-lymphocyte levels was noted (M = 16.7, SD = 2.6); however, the count remained 37.2% lower than that of the control group (M = 26.6, SD = 3.0; *p* = 0.0025). In contrast, the level of CD4^+^CD25^+^ T cells continued to decline (M = 3.4, SD = 0.4), suggesting a persistent state of immunodeficiency and continued deficiency in immunoregulatory function.

*Late stage of acute pneumonia* (*day 14*). By day 14, CD4^+^ T-lymphocyte levels (M = 18.2, SD = 6.5) remained significantly below control values (*p* = 0.0172), indicating incomplete immune recovery. The early-phase depletion of CD4^+^ T cells may be attributed to their active migration into pulmonary tissue, apoptosis, cytokine-induced stress, and immune exhaustion. While this response may be part of the physiological immune process, excessive depletion can impair adaptive immunity and increase the risk of complications.

Animals in the AP group displayed clear signs of immune dysfunction, which was characterized by sustained CD4^+^ T-cell deficiency and the inadequate activation of immunoregulatory mechanisms. The restoration of immune parameters proceeded slowly, reinforcing the importance of immunomodulatory interventions to accelerate immune regeneration and stabilize immune homeostasis. The restoration of these key immune indices is a critical objective of correcting immune dysregulation. The present study was conducted to assess the dynamics of CD4^+^ T-cell populations and their subsets in response to the immunomodulatory agents (***o*-Fph**)**PPhβCD** and PO.

*Effects of (**o-Fph**)**PPhβCD** and PO on the Immunological Profiles of the Spleen During Acute Pneumonia.*

*Day 3*

*CD4^+^ T cells*: Treatment with (***o*-Fph**)**PPhβCD** and PO resulted in a substantial increase in CD4^+^ T-cell levels (32.8 ± 6.0 and 32.8 ± 10.8, respectively), exceeding the control values. This indicates pronounced immunomodulatory activity by both agents.

*CD4^+^CD25^+^ T cells*: In the AP group, CD4^+^CD25^+^ levels were reduced by 27% compared to controls (4.0 ± 1.5 vs. 5.5 ± 1.6). A further decrease was observed in the AP/(***o*-Fph**)**PPhβCD** group (1.6 ± 0.4), and an even more marked reduction occurred in the AP/PO group (0.9 ± 0.5). These results suggest the suppression of the regulatory arm of the immune response during the early phase of treatment.

*CD4^+^CD25^+^FoxP3^+^ T cells*: In the AP group, CD4^+^CD25^+^FoxP3^+^ regulatory T-cell levels were significantly lower than in the control group, indicating the marked suppression of regulatory T-cell function. Further reductions were observed under the influence of both (***o*-Fph**)**PPhβCD** and PO, suggesting a decrease in immune tolerance and enhanced activation of the effector immune response. The observed early reduction in CD4^+^CD25^+^FoxP3^+^ cells during pneumonia may be attributed to hyperactivation of the inflammatory response, cytokine-induced stress, apoptosis, and redistribution of these cells to the site of infection. While this may enhance effector immunity during the initial stages of the disease, it may also compromise the long-term control of inflammation and contribute to tissue damage [20]. According to Reference [21], during the early days of pneumonia, a portion of circulating Tregs may migrate into the lungs, where they participate in local immune regulation. This migration may account for their reduced levels in peripheral organs but does not necessarily imply complete depletion.

*Day 7*

*CD4^+^ T cells*: In the AP group, the partial restoration of CD4^+^ levels was observed (16.7 ± 2.6), although the count remained 37% below that of the control group. Treatment with (***o*-Fph**)**PPhβCD** (17.7 ± 2.6) and PO (19.0 ± 3.1) did not result in a statistically significant increase compared to the AP group, although PO showed a trend toward greater efficacy.

*CD4^+^CD25^+^ T cells*: The CD4^+^CD25^+^ subset continued to decline in the AP group (3.4 ± 0.4 vs. 5.5 ± 1.6 in the control group, *p* < 0.05). Treatment with (***o*-Fph**)**PPhβCD** led to an increase (3.0 ± 0.4), although the level remained below control values. PO exhibited a weaker effect (2.2 ± 0.5).

*CD4^+^CD25^+^FoxP3^+^ T cells*: In the AP group, the level of regulatory T cells continued to decline. Both (***o*-Fph**)**PPhβCD** and PO further suppressed this subset, with (***o*-Fph**)**PPhβCD** producing a more pronounced reduction. This suggests its potential to enhance effector immune mechanisms.

*Day 14*

*CD4^+^ T cells*: In the AP group, CD4^+^ levels remained lower than in controls (18.2 ± 6.5 vs. 26.6 ± 3.0). Treatment with (***o*-Fph**)**PPhβCD** resulted in a significant increase (22.8 ± 4.9), while PO had a more modest effect (18.8 ± 3.7).

*CD4^+^CD25^+^ T cells*: The AP group showed a persistent reduction (3.3 ± 0.6), indicating a continued immunodeficient state. Treatment with (***o*-Fph**)**PPhβCD** led to a notable increase (4.2 ± 0.8), whereas PO had a lesser impact (2.2 ± 0.8).

*CD4^+^CD25^+^FoxP3^+^ T cells*: A marked decrease in regulatory T cells was observed in the AP group, suggesting an ongoing immune imbalance. Both (***o*-Fph**)**PPhβCD** and PO maintained low levels of this subset, with (***o*-Fph**)**PPhβCD** inducing a more substantial reduction, consistent with enhanced activation of the effector immune compartment.

## 3. Materials and Methods

### 3.1. Chemical Experimental Part

#### 3.1.1. Synthesis and Structural Characterization of Chemical Compounds

The β-cyclodextrin used in the synthesis was obtained from Sigma-Aldrich (St. Louis, MO, USA, CAS No. 7585-39-9). The compound dimethyl[(4-benzhydrylpiperazin-1-yl)(2-fluorophenyl)methyl]phosphonate was synthesized, purified, and structurally identified in accordance with the procedure described in Reference [16]. The progress of the reactions and the purity of the resulting compounds were monitored by thin-layer chromatography (TLC) on aluminum oxide (Al_2_O_3_) plates. Visualization of the analyte spots was performed using iodine vapor. The mobile phase consisted of a benzene–ethanol mixture in a 3:1 ratio. Elemental composition was confirmed by elemental analysis using a Rapid Micro N Cube elemental analyzer. IR spectra were recorded on a Bruker Alpha-P ATR-FTIR spectrometer (diamond crystal, Bruker, Billerica, MA, USA) over the spectral range of 400–4000 cm^−1^ using KBr pellet techniques. The proton (^1^H) and carbon (^13^C) NMR spectra were acquired with a JNM-ECZ 600R spectrometer (JEOL, Tokyo, Japan) operating at 600 MHz for ^1^H and 150 MHz for ^13^C nuclei. Deuterated dimethyl sulfoxide (DMSO-*d*_6_) was used as the solvent. TMS was used as an internal standard in the ^1^H NMR spectra. In the ^13^C NMR spectra, the chemical shift of the solvent (DMSO-*d*_6_, at 39.52 ppm relative to TMS) was used as an internal standard.

#### 3.1.2. Inclusion Complex of Dimethyl[(4-benzhydrylpiperazin-1-yl)(2-fluorophenyl)methyl]phosphonate with β-Cyclodextrin ((**o-Fph**)**PPhβCD**)

**Scheme 2:**
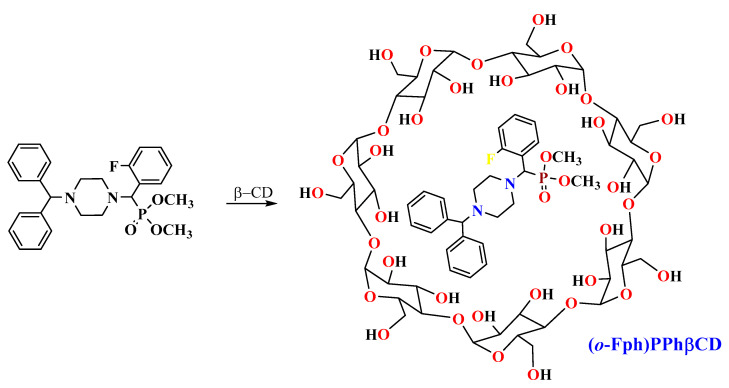
Scheme of complexation with β-cyclodextrin.

To prepare the inclusion complex of dimethyl[(4-benzhydrylpiperazin-1-yl)(2-fluorophenyl)methyl]phosphonate with β-cyclodextrin, 0.94 g (0.002 mol) of the phosphonate was dissolved in 45 mL of ethyl alcohol and mixed with 2.27 g (0.002 mol) of β-cyclodextrin dissolved in 50 mL of distilled water. The resulting mixture was subjected to ultrasonic treatment for 30 min at 50 °C and then transferred to a drying oven, where the solvents (ethanol and water) were evaporated at 50–55 °C. This process yielded 3.07 g (95.6%) of the target complex as a white powder, which decomposed upon melting at temperatures above 250 °C (Scheme 2).

Elemental analysis of the complex, with the molecular formula C_68_H_100_FN_2_O_38_P, provided the following calculated values: carbon (C) 50.94%, hydrogen (H) 6.29%, fluorine (F) 1.18%, nitrogen (N) 1.75%, and phosphorus (P) 1.93%. The experimentally determined values were as follows: carbon (C) 51.01%, hydrogen (H) 6.43%, fluorine (F) 1.20%, nitrogen (N) 1.69%, and phosphorus (P) 1.90%.

For details of IR (KBr, ν, cm^−1^), see Figures S1–S5.

For details of ^1^H NMR and ^13^C NMR (DMSO-*d*_6_, δ, ppm), see Figures S6–S9.

### 3.2. Biological Experimental Part

#### 3.2.1. Experimental Animals

The study was conducted on male rats weighing 180–220 g, which were housed in a specialized animal facility in compliance with international standards [22] and in accordance with the Rules for Conducting Biomedical Experiments, Preclinical (Non-clinical), and Clinical Studies, and Requirements for Preclinical and Clinical Facilities approved by Order No. 142 of the Minister of Health of the Republic of Kazakhstan, dated 2 April 2018 (registered with the Ministry of Justice of the Republic of Kazakhstan on 17 April 2018, No. 16768) [23]. Experimental groups were formed using random sampling based on body weight, and the animals were identified and allocated to cages according to their assigned groups. Decapitation was performed under general anesthesia using Zoletil-Xylazine [24].

Four experimental series were conducted, each involving 30 male rats per group, except for the control group, which included 10 animals. Evaluations were carried out on days 3, 7, and 14 post-induction. The groups were designated as follows: C—control, AP—acute pneumonia, (***o*-Fph**)**PPhβCD**—test compound group, and PO—Polyoxidonium [25,26], which is a clinically approved immunomodulatory reference drug.

The study assessed the proportions of CD4^+^, CD4^+^CD25^+^ (activated T cells), and CD4^+^CD25^+^FoxP3^+^ (regulatory T cells) using flow cytometry (see Supplementary Materials).

#### 3.2.2. Experimental Design

**Figure 4:**
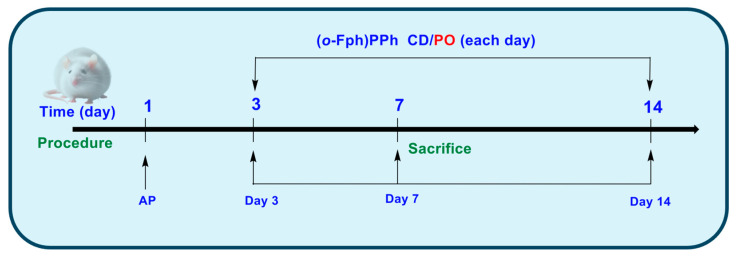
Experimental design.

Group C (Control) comprised intact animals that did not receive any treatment or intervention. Group AP comprised animals in which acute pneumonia was experimentally induced [27]. In Group AP/(***o*-Fph**)**PPhβCD**, following the induction of acute pneumonia, the animals received (***o*-Fph**)**PPhβCD**. In Group AP/PO, following the induction of acute pneumonia, the animals were treated with PO for 10 consecutive days (Figure 4).

Modeling of acute pneumonia in rats [27] in the AP, AP/(***o*-Fph**)**PPhβCD**, and AP/PO groups was performed under general anesthesia, using Zoletil-Xylazine at doses of 20–40 mg/kg and 5–10 mg/kg, respectively, following intramuscular administration. Prior to anesthesia, animals received premedication with atropine at 0.5–1 mg/kg, also administered intramuscularly. Acute lung injury was induced in our study by the intravenous administration of an oleic acid suspension, a well-established non-infectious model of acute pneumonia that replicates the key features of alveolar-capillary barrier disruption and inflammatory infiltration. Specifically, a suspension of oleic acid in an albumin–saline solution was prepared by the vigorous mixing of oleic acid in a 0.1 mg/mL (*w*/*v*) solution of bovine serum albumin in sterile physiological saline at a volumetric ratio of 1:5. For model induction, 0.25 mL of this suspension was administered via intravenous injection. In contrast, the control animals (Group C) received an equivalent volume of physiological saline. Following the procedure, all animals were returned to their cages and monitored until their full recovery from anesthesia.

In each experimental series, the animals were euthanized on days 3, 7, and 14 after pneumonia induction. Euthanasia was performed under Zoletil-Xylazine anesthesia, with prior atropine premedication [23]. At each time point, 30 rats per group and 10 control animals (one-time collection) were sacrificed. The spleens were collected for immunological analysis using flow cytometry (Attune™ NxT, Thermo Fisher Scientific, Waltham, MA, USA) [13] (see Supplementary Materials).

#### 3.2.3. Statistical Analysis

All experiments were performed with a minimum of ten replicates. Statistical analysis was conducted using GraphPad Prism 10 software. Graphs and figures present data as arithmetic means (M) ± standard deviation (SD), with the corresponding values summarized in Table 3.

The statistical significance of the differences between the mean values from two experimental groups was calculated using the TTEST function. Differences were considered not significant if the null hypothesis probability exceeded 5% (*p* > 0.05). The results of the comparative analysis are presented in Table 4.

## 4. Study Limitations

Several limitations of this study should be acknowledged. First, cytokine profiling, hematological parameters, and blood gas analysis were not included in this manuscript, as they are part of an ongoing parallel investigation and will be reported in a separate publication focused on systemic immunological and biochemical responses. Second, while the 14-day observation period may appear short, it is in fact well-suited to the oleic acid-induced pneumonia model, which replicates the rapid progression of acute respiratory distress. This timeframe allows for the assessment of both acute inflammatory damage and early reparative processes, thereby enabling a comprehensive evaluation of treatment efficacy. Third, although quantitative histological scoring is not presented here, due to suboptimal image resolution, a microscopic evaluation of lung tissue was performed and is now included to qualitatively support the immunological findings. These limitations, while notable, do not detract from the core conclusion that (***o*-Fph**)**PPhβCD** exhibits significant immunomodulatory activity during acute pneumonia.

## 5. Conclusions

This study successfully synthesized and structurally confirmed a β-cyclodextrin inclusion complex of dimethyl[(4-benzhydrylpiperazin-1-yl)(2-fluorophenyl)methyl]phosphonate ((***o*-Fph**)**PPhβCD**) with a high yield and high stability. Biological evaluation in a rat model of acute pneumonia demonstrated that the complex exerts significant immunomodulatory effects, restoring CD4^+^ and CD4^+^CD25^+^ T-cell populations while reducing immunosuppressive CD4^+^CD25^+^FoxP3^+^ regulatory T cells. Compared to the reference agent Polyoxidonium (PO), (***o*-Fph**)**PPhβCD** provided more sustained immune correction, particularly during the late phase of disease progression. These results suggest that (***o*-Fph**)**PPhβCD** enhances adaptive immunity by supporting effector responses and may serve as a viable adjunct or alternative to conventional immunomodulators in the treatment of acute lung inflammation. Further investigation, including cytokine profiling and histological scoring, is warranted to elucidate its full therapeutic potential.

## Figures and Tables

**Figure 2:**
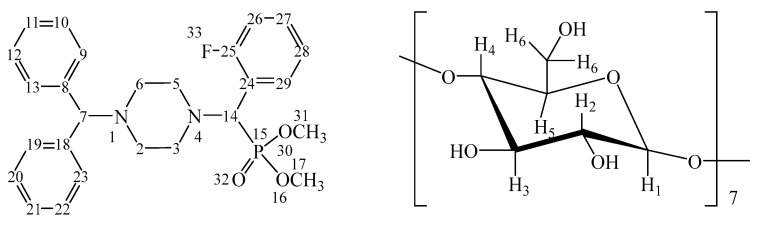
Numbering of atoms in (***o*-Fph**)**PPhβCD** (calculated separately for the aminophosphonate and oligosaccharide units).

**Table 1:** IR Absorption band values (cm^−1^) of β-CD, (*o*-Fph)PPh, and (***o*-Fph**)**PPhβCD**.

Groups	β-CD (Figure S1)	(*o*-Fph)PPhβCD (Figure S2)	(*o*-Fph)PPh (Figure S4) [16]	Note
C–H ar (C–OH)	– (1619.2–1639.0)	1641.9 wide	1585.5, 1612.4, 1699.1	Changes in absorption in the complex compared to both β-CD and (*o*-Fph)PPh
P–C	–	759.0	759.2	Decreased absorption band intensity in the spectrum of the (***o*-Fph**)**PPhβCD**
P=O (C–O–C)	(1028.0, 1080.3)	1079.4 (1029.7)	1052.3	Shift in the P=O bond absorption (from 1052.3 to 1079.4 cm^−1^)
C–N (C–O–C)	(1157.9)	1156.5 (1156.5)	1187.4	Shift in the C–N bond absorption (from 1187.4 to 1156.5 cm^−1^)
C–F (C–O–C)	(1250.3)	1231.0	1227.6	Shift in the C–F bond absorption (from 1227.6 to 1031.0 cm^−1^)

**Table 2:** Chemical shift values (δ, ppm) of ^1^H and ^13^C nuclei for (*o*-Fph)PPh and β-CD in their free state (δ_0_) and in the IC (***o*-Fph**)**PPhβCD** (δ).

Atom No.	CH_x_ Group	δ_0_, ppm (CDCl_3_)		δ, ppm (DMSO-*d*_6_)		∆δ = δ − δ_0_	
		^1^H	^13^C	^1^H	^13^C	^1^H	^13^C
(***o*-Fph**)**PPhβCD**							
2ax,6ax	CH_2_	2.34 s	52.23	2.27–2.45 m	52.25	0.02	0.02
2eq,6eq		2.34 s		2.27–2.45 m		0.02	
3ax,5ax	CH_2_	2.52–2.80 m	52.91, 54.70	2.48–2.77 m	52.95, 54.74	−0.03	0.04 0.04
3eq,5eq		2.52–2.80 m		2.48–2.77 m		−0.03	
7	CH	4.43–4.47 m	75.36	4.38–4.43 m	75.48	−0.04	0.12
8,18	=C<	–	142.19	–	143.25	–	1.06
9,13	–CH=	7.18–7.55 m	129.09	7.18–7.55 m	129.01	0	−0.08
10,12	–CH=	7.1–7.55 m	127.64	7.18–7.55 m	128.04	0	0.40
11,21	–CH=	7.18–7.55 m	127.54	7.18–7.55 m	127.36	0	−0.18
14	CH	4.32–4.38 m	58.25	4.19–4.34 m	57.90	0.10	−0.35
19,23	–CH=	7.18–7.55 m	129.09	7.18–7.55 m	129.01	0	−0.08
20,22	–CH=	7.18–7.55 m	127.64	7.18–7.55 m	128.04	0	0.40
17,31	CH_3_O–	3.37–3.40 m, 3.75–3.78 m	56.50	3.38–3.40 m, 3.76–3.77 m	56.40, 56.89	0 0	−0.10 −0.39
24	=C<	–	115.92, 117.34	–	115.89, 118.99	–	−0.03 1.65
25	–FC=	–	160.03, 162.46	–	160.44, 162.11	–	0.41 −0.35
26	–CH=	7.11–7.15 m	115.92, 117.34	7.08–7.12 m	115.89, 118.99	−0.03	−0.03 1.65
27	–CH=	7.81–7.92 m	132.07	7.68–7.71 m	132.29	−0.17	0.22
28	–CH=	7.11–7.15 m	124.82	7.08–7.12 m	124.68	−0.03	−0.14
29	–CH=	7.18–7.55 m	129.09	7.18–7.55 m	130.83	0	1.74
Β-CD							
1	>CH	4.76 s	102.42	4.79 s	102.70	0.01	0.38
2	>CH	3.22–3.27 m	72.86	3.25–3.28 m	73.21	0.02	0.35
3	>CH	3.48–3.55 m	73.54	3.56–3.58 m	73.75	0.05	0.21
4	>CH	3.27–3.29 m	82.01	3.30–3.33 m	82.22	0.04	0.21
5	>CH	3.49 s	72.51	3.51–3.53 m	72.46	0.03	−0.05
6	CH_2_	3.55–3.57 m	60.42	3.60–3.63 m	60.62	0.06	0.20

**Table 3:** Mean values and standard deviations of T lymphocyte populations across the experimental groups.

Groups	M ± SD		
	Day 3	Day 7	Day 14
	CD4^+^		
C (control)	26.6 ± 3.0	26.6 ± 3.0	26.6 ± 3.0
AP (acute pneumonia)	11.2 ± 1.8	16.7 ± 2.6	18.2 ± 6.5
AP/(***o*-Fph**)**PPhβCD**	32.8 ± 6.0	17.7 ± 2.6	22.8 ± 4.9
AP/PO	32.8 ± 10.8	19.0 ± 3.1	18.8 ± 3.7
	CD4^+^CD25^+^		
C (control)	5.5 ± 1.6	5.5 ± 1.6	5.5 ± 1.6
AP (acute pneumonia)	4.0 ± 1.5	3.4 ± 0.4	3.3 ± 0.6
AP(***o*-Fph**)**PPhβCD**	1.6 ± 0.4	3.0 ± 0.4	4.2 ± 0.8
AP/PO	0.9 ± 0.1	2.2 ± 0.5	2.2 ± 0.8

**Table 4:** Group comparisons (*t*-test *p*-values).

Group Comparisons (*t*-Test *p*-Values)	*p*			Conclusion
	Day 3	Day 7	Day 14	
CD4^+^				
C vs. AP	0.0001	0.0025	–	A significant reduction in CD4^+^ levels was observed in the AP group—by 137.5% (day 3) and 59.3% (day 7).
C vs. (***o*-Fph**)**PPhβCD**	–	0.0043	–	The difference in CD4^+^ levels in the AP/(***o*-Fph**)**PPhβCD** group was statistically significant but moderate (16.7%).
C vs. PO	–	0.0124	0.0172	A slight decrease in CD4^+^ was noted in the AP/PO group—40.0% (day 7) and 41.5% (day 14).
AP vs. (***o*-Fph**)**PPhβCD**	0.0005	–	–	A substantial threefold difference was observed in favor of (***o*-Fph**)**PPhβCD**.
AP vs. PO	0.0076	–	–	A substantial threefold difference was observed in favor of PO.
(***o*-Fph**)**PPhβCD** vs. PO	–	–	–	No significant difference was detected.
CD4^+^CD25^+^				
C vs. AP	–	0.0442	0.0462	A statistically significant reduction of over 60% in CD4^+^CD25^+^ levels was observed in the AP group (days 7 and 14)
C vs. (***o*-Fph**)**PPhβCD**	0.0035	0.0276	–	A substantial difference in CD4^+^CD25^+^ levels was noted in the AP/(***o*-Fph**)**PPhβCD** group compared to the control—3.4-fold (day 3) and 1.8-fold (day 7).
C vs. PO	0.0017	0.0090	0.0114	In the AP/PO group, a significant difference in CD4^+^CD25^+^ levels compared to the control was observed—6.1-fold (day 3) and 2.5-fold (day 7).
AP vs. (***o*-Fph**)**PPhβCD**	0.0197	–	–	Compared to the AP group, CD4^+^CD25^+^ levels in the AP/(***o*-Fph**)**PPhβCD** group were significantly higher—2.5-fold (day 3).
AP vs. PO	0.0072	0.0098	–	In the AP/PO group, CD4^+^CD25^+^ levels were 4.4-fold higher (day 3) and showed a minor difference of 1.5-fold (day 7) compared to the AP group.
(***o*-Fph**)**PPhβCD** vs. PO	–	0.0511	0.0144	A notable advantage was seen in favor of (***o*-Fph**)**PPhβCD** over PO—36.4% (day 7) and 91.0% (day 14).
CD4^+^CD25^+^FoxP3^+^				
C vs. AP	0.0212	0.0449	–	A significant 1.8-fold reduction in CD4^+^CD25^+^FoxP3^+^ levels was observed in the AP group compared to the control (days 7 and 14).
C vs. (***o*-Fph**)**PPhβCD**	–	0.0337	–	A twofold decrease in CD4^+^CD25^+^FoxP3^+^ levels was noted in the AP/(***o*-Fph**)**PPhβCD** group relative to the control (day 7).
C vs. PO	0.0034	0.0403	–	A marked reduction of twofold or more in CD4^+^CD25^+^FoxP3^+^ levels was observed in the AP group compared to the control (days 3 and 7).
AP vs. (***o*-Fph**)**PPhβCD**	–	–	–	No significant difference was observed.
AP vs. PO	0.0180	–	–	A twofold reduction in CD4^+^CD25^+^FoxP3^+^ levels was recorded in the AP/PO group compared to the AP group (day 3).
(***o*-Fph**)**PPhβCD** vs. PO	00052	–	–	A significant 2.7-fold difference in favor of (***o*-Fph**)**PPhβCD** over PO was observed.

## Data Availability

The datasets used and/or analyzed during the present study are available from the corresponding author upon reasonable request.

## Supplementary Materials

The following supporting information can be downloaded at https://www.mdpi.com/article/10.3390/molecules30132741/s1: FLOW CYTOMETRY; Table S1: Antibodies and fluorochromes used for immunophenotyping of splenic subpopulations in rats treated with the complex; Figure S1: IR (KBr, ν, cm^−1^) spectrum of β-cyclodextrin (β-CD); Figure S2: IR (KBr, ν, cm^−1^) spectrum of complex of dimethyl[(4-benzhydrylpiperazin-1-yl)(*o*-fluorophenyl)methyl]phosphonate with β-CD ((***o*-Fph**)**PPhβCD**); Figure S3: IR (KBr, ν, cm^−1^) spectrum of dimethyl[(4-benzhydrylpiperazin-1-yl)(*o*-fluorophenyl)methyl]phosphonate ((*o*-Fph)PPh) [16]; Figure S4: Comparative IR (KBr, ν, cm^−1^) spectrum of (***o*-Fph**)**PPhβCD** (blue) and βCD (orange); Figure S5: Comparative IR (KBr, ν, cm^−1^) spectrum of (***o*-Fph**)**PPhβCD** (blue) and (*o*-Fph)PPh (red) [16]; Figure S6: ^1^H NMR (399.78 MHz, DMSO-*d*_6_) spectrum of **(*o*-Fph)PPh**; Figure S7: ^13^C NMR (100.53 MHz, DMSO-*d*_6_) spectrum of (*o*-Fph)PPh; Figure S8: ^1^H-^1^H COSY spectrum of of (*o*-Fph)PPh; Figure S9: ^1^H NMR (399.78 MHz, DMSO-*d*_6_) spectrum of (***o*-Fph**)**PPhβCD**; Figure S10: ^13^C NMR (100.53 MHz, DMSO-*d*_6_) spectrum of (***o*-Fph**)**PPhβCD**; Figure S11: ^1^H-^1^H COSY spectrum of of (***o*-Fph**)**PPhβCD**; Figure S12: ^1^H-^13^C HMBC spectrum of (***o*-Fph**)**PPhβCD**.
