# Supplementary material for: Targeted Restoration of T-Cell Subsets by a Fluorinated Piperazine Derivative β-Cyclodextrin Complex in Experimental Pulmonary Inflammation
Source: Molecules. 2025 Jun 25;30(13):2741. doi: 10.3390/molecules30132741 (PMC12250881; doi:10.3390/molecules30132741)
Supplement: Supplementary file 1 [file molecules-30-02741-s001.zip › molecules-3702589-supplementary.pdf]

Supplementary Materials

FLOW CYTOMETRY

(1) Sample preparation and staining of splenocytes for flow cytometric analysis

Spleen isolation and processing: Rat spleens, approximately 0.5 × 2 cm in size, were harvested and immersed in 0.5 mL of ice-cold physiological saline. The spleen tissues were mechanically dissociated and homogenized in an additional 4.5 mL of ice-cold physiological saline. The resulting cell suspension was filtered through 70 μm nylon mesh filters to remove tissue debris, followed by centrifugation at 400 × g for 5 minutes to pellet the cells. Red blood cells were lysed using 2 mL of High-Yield Lyse reagent. The splenocyte pellet was resuspended in the reagent and incubated for 10 minutes at room temperature. Subsequently, the cells were washed twice with 2 mL of physiological saline to remove residual lysing agent and then resuspended in 100 μL of cold physiological saline. Surface staining of splenocytes was performed using fluorochrome-conjugated monoclonal antibodies specific for cell surface markers, following the protocols provided by the antibody manufacturers. After staining, cells were washed with 2 mL of physiological saline, fixed, and resuspended in 500 μL of 0.9% physiological saline. Fluorescence Minus One (FMO) controls were used to assess and correct for nonspecific fluorescence. Stained cell suspensions were analyzed using the Attune™ NxT Flow Cytometer (Thermo Fisher Scientific). Quantitative assessment focused on the proportions of major T lymphocyte subsets, including $CD4^+$, $CD4^+CD25^+$, and $CD4^+FoxP3^+$ populations (as summarized in the accompanying table). Data analysis was conducted using the Attune™ NxT Cytometric Software, version 6.0.1 (Thermo Fisher Scientific).

Table S1: Antibodies and fluorochromes used for immunophenotyping of splenic subpopulations in rats treated with the complex

Category Code	Antibody	Fluorochrome	Manufacturer	Clone
46-0040-82	CD4	PerCP-eFluor™ 710	eBioscience™	OX-35
17-0390-82	CD25	APC	eBioscience™	OX-39
11-5773-82	FOXP3	FITC	eBioscience™	FJK-16s
MA517540	KLRB1	PE	eBioscience™	10-78

(2) T-lymphocyte gating strategy for flow cytometric analysis

1. Primary lymphocyte gating (FSC vs SSC):

The initial step in flow cytometric analysis involved gating based on light scatter properties to identify the lymphocyte population. Forward Scatter (FSC) was utilized to estimate cell size, while Side Scatter (SSC) provided information on the internal complexity and granularity of the cells.

Lymphocytes were characterized by their relatively small size and low granularity, which enabled effective discrimination from other peripheral blood components such as monocytes, neutrophils, and erythrocyte debris. This gating strategy ensured a high-purity lymphocyte subset for subsequent analyses.

2. Delineation of T-lymphocyte subpopulations

Following the establishment of the lymphocyte gate, subpopulation delineation was performed using fluorescently labeled monoclonal antibodies targeting lineage-specific surface markers.

Specifically, $CD4^+$ T-lymphocytes were identified using anti-CD4 monoclonal antibodies conjugated to appropriate fluorochromes. This allowed for the accurate discrimination and quantification of the helper T-cell subset within the total lymphocyte pool.

3. Identification of regulatory T Cells (Tregs)

Within the $CD4^+$ T-lymphocyte population, regulatory T cells (Tregs) were further delineated based on the expression of key phenotypic markers:

- **$CD4^+CD25^+$ Tregs:** A subset of $CD4^+$ cells exhibiting high expression of CD25, the α-chain of the interleukin-2 receptor, was gated to identify putative regulatory T cells. Elevated CD25 expression is a hallmark feature of this immunoregulatory subset.
- **$CD4^+FoxP3^+$ Tregs:** To confirm the regulatory phenotype, intracellular staining for the transcription factor FoxP3 was performed. FoxP3 is a definitive molecular marker critical for the development and function of regulatory T cells and was used to validate the Treg identity within the $CD4^+$ population.

4. Gating quality control

The accuracy and consistency of gating strategies were validated using control samples with well-characterized immunophenotypes. Additionally, fluorescence signal distributions were systematically evaluated to exclude nonspecific antibody binding and autofluorescence artifacts.

The rigorously gated subpopulations were subsequently utilized for downstream analyses to assess their functional attributes and phenotypic profiles.

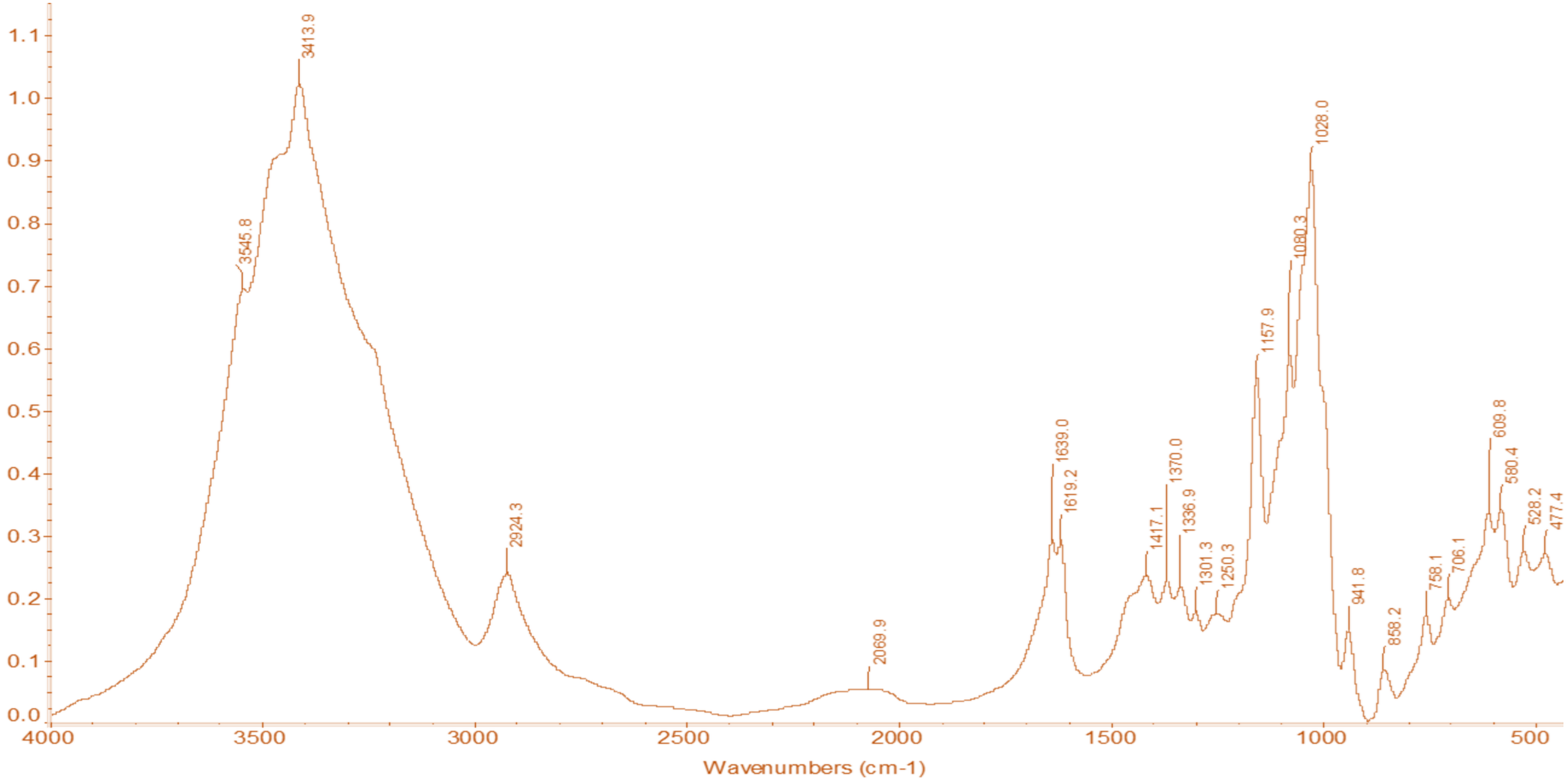

Figure S1. IR (KBr, ν, cm^{-1}) spectrum of β-cyclodextrin (β-CD).

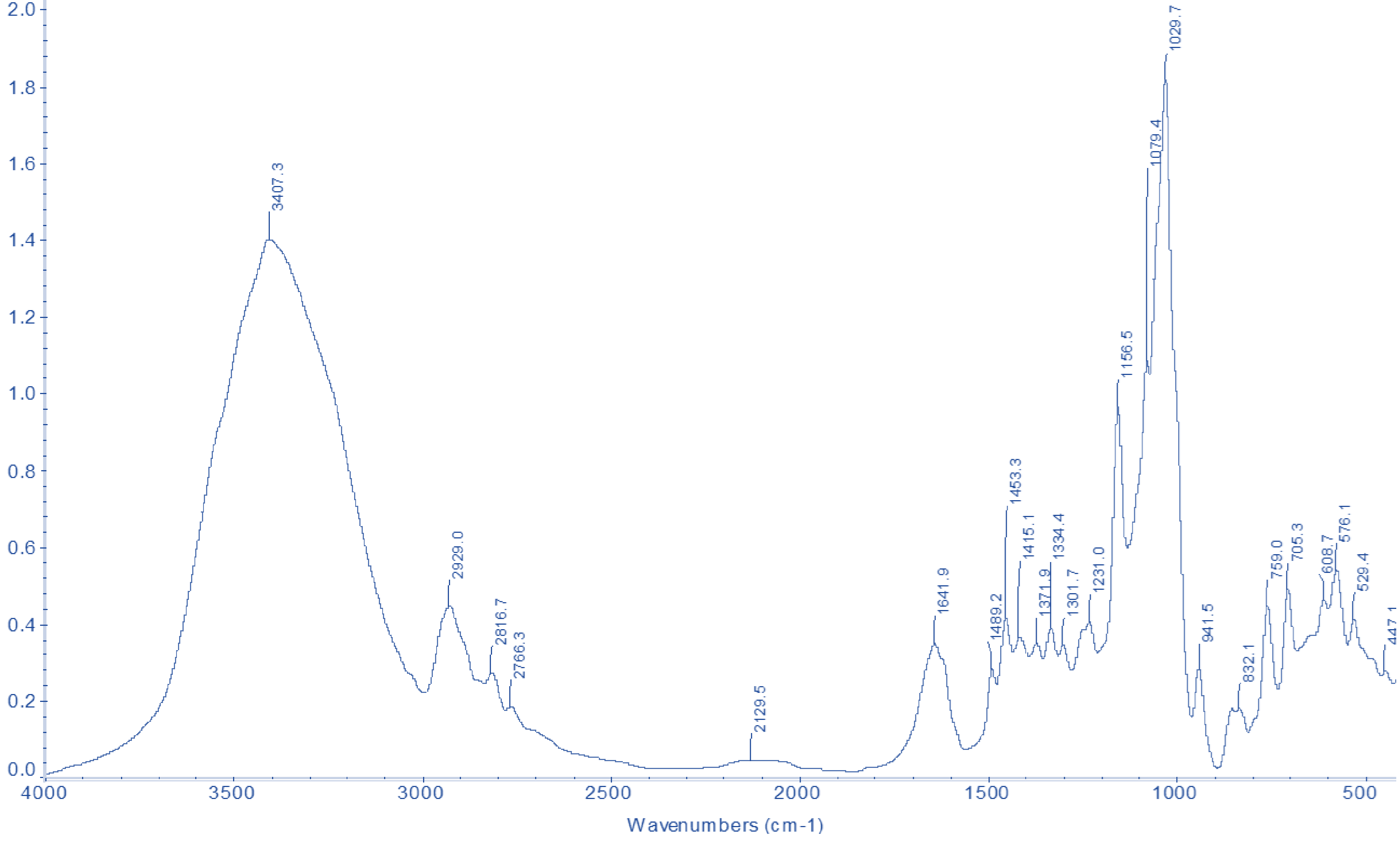

Figure S2. IR (KBr, ν, cm^{-1}) spectrum of complex of dimethyl[(4-benzhydrylpiperazin-1-yl)(*o*-fluorophenyl)methyl]phosphonate with β-CD ((***o*-Fph**)**PPhβCD**).

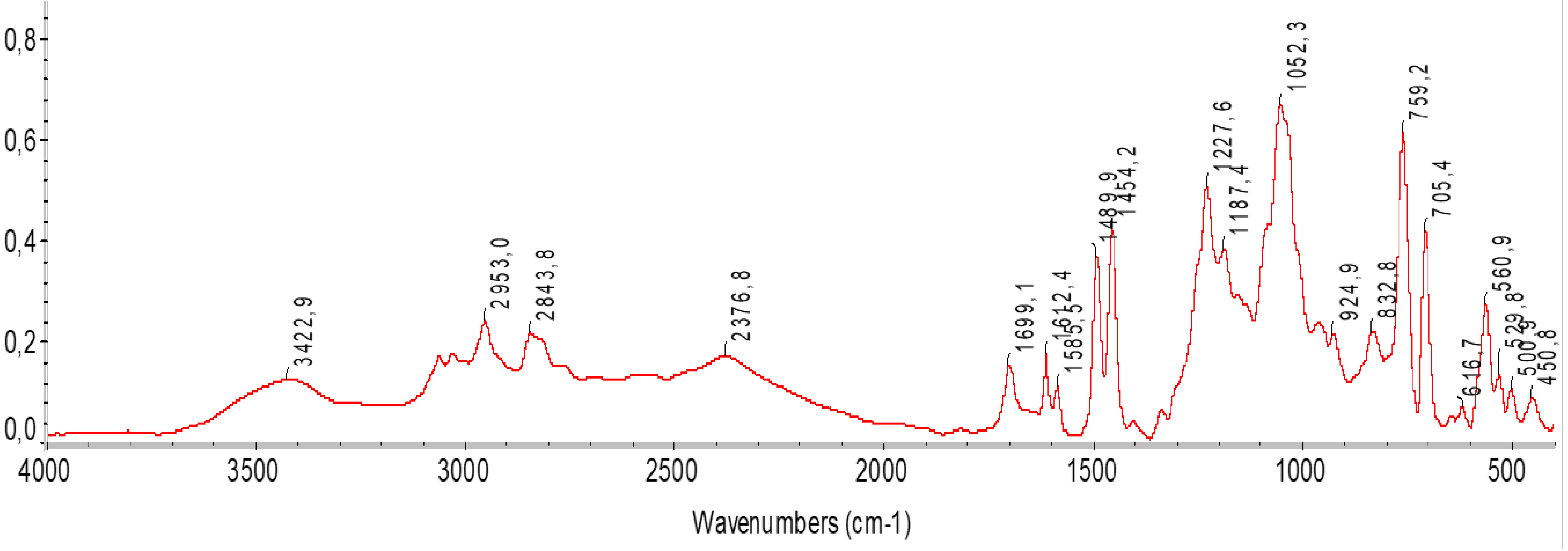

Figure S3. IR (KBr, ν, cm^{-1}) spectrum of dimethyl[(4-benzhydrylpiperazin-1-yl)(*o*-fluorophenyl)methyl]phosphonate ((*o*-Fph)PPh)[16].

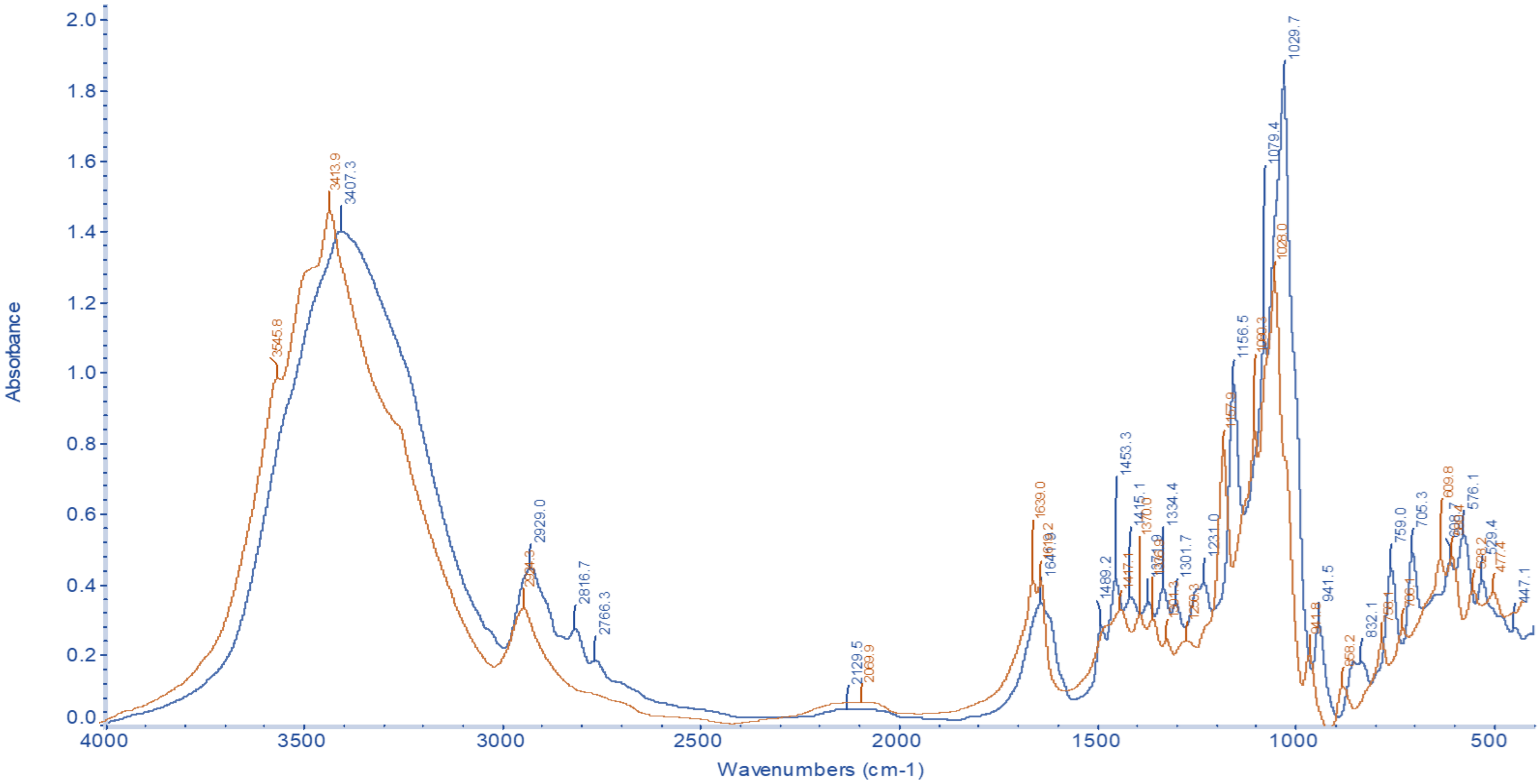

Figure S4. Comparative IR (KBr, ν, cm-1) spectrum of (***o*-Fph**)**PPhβCD** (blue) and β-CD (orange).

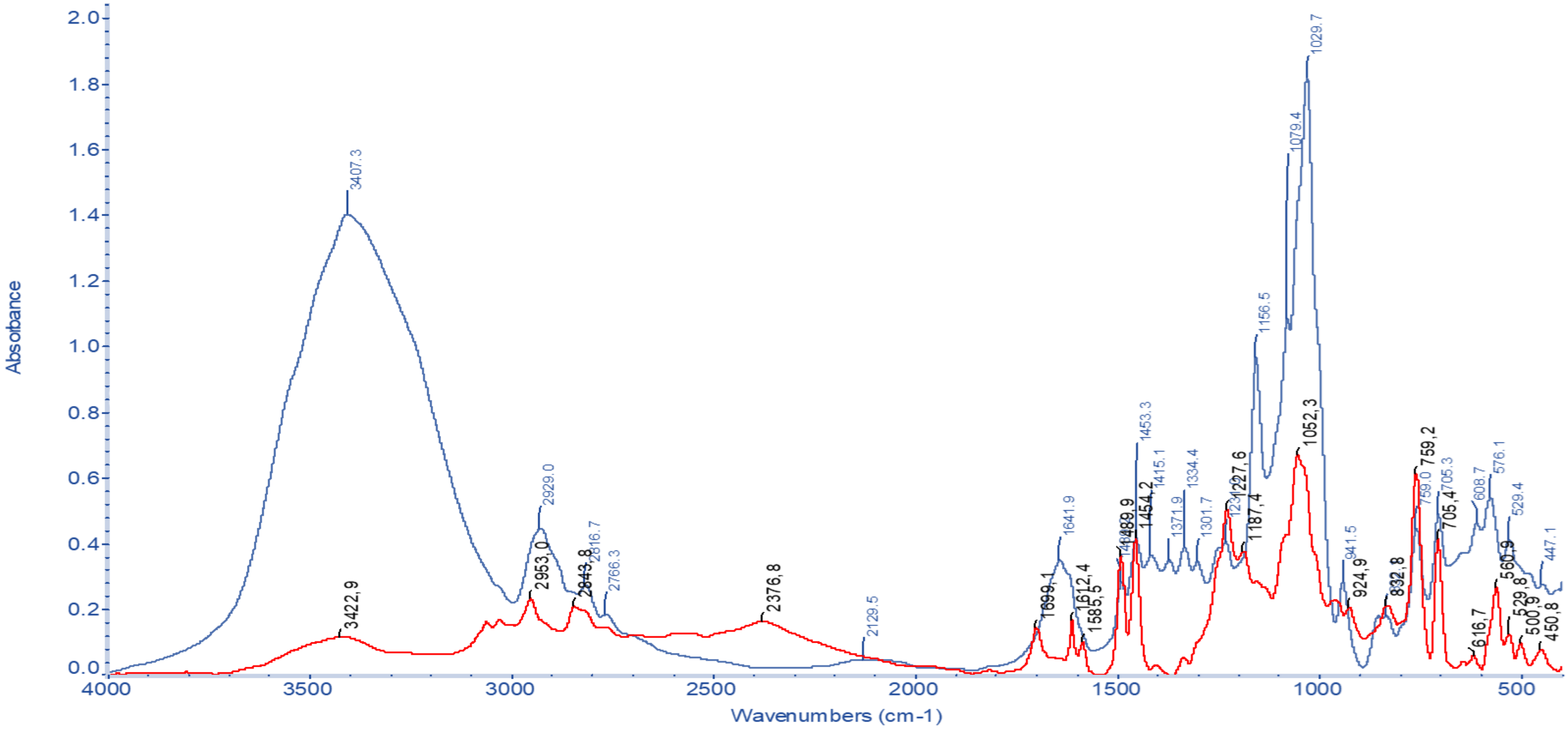

Figure S5. Comparative IR (KBr, ν, cm^{-1}) spectrum of (***o*-Fph**)**PPhβCD** (blue) and (*o*-Fph)PPh (red) [16].

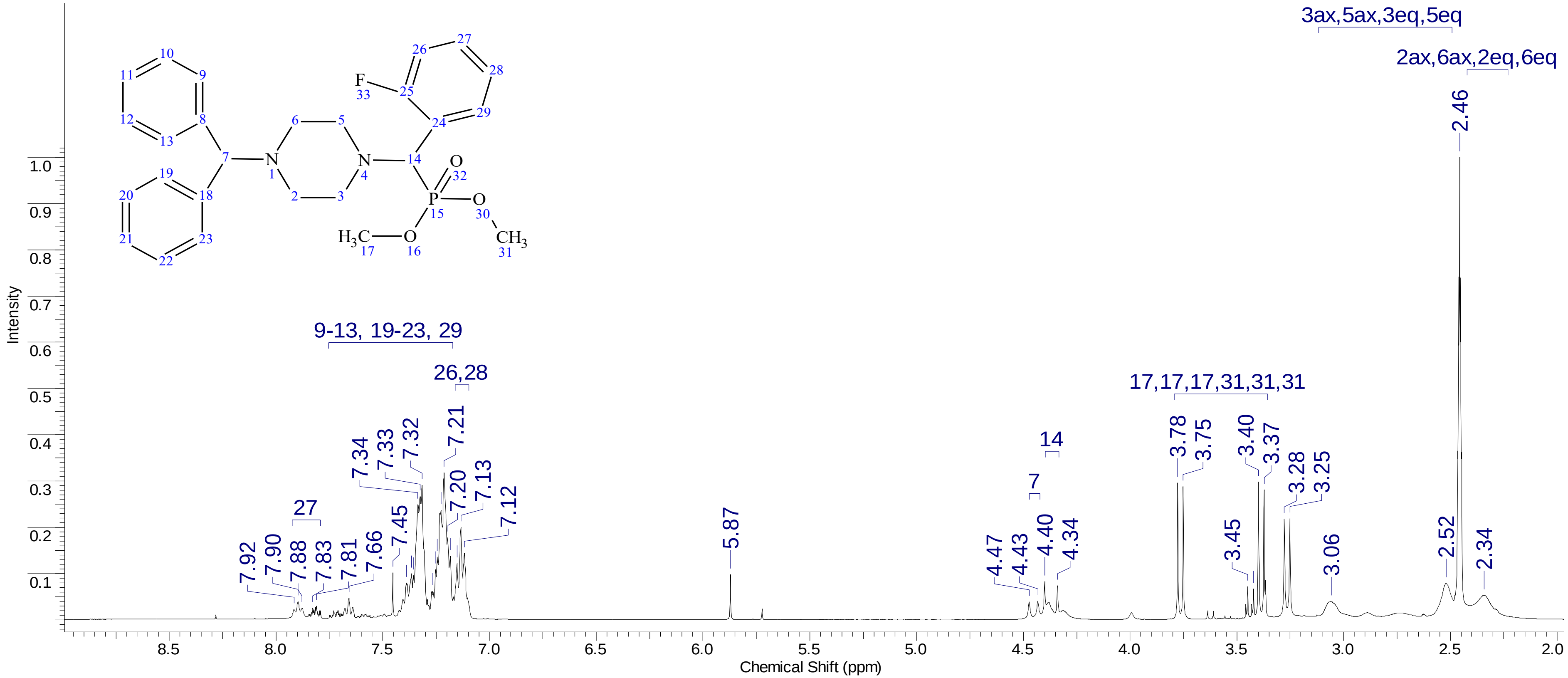

Figure S6. ^{1}H NMR (399.78 MHz, DMSO-d_6) spectrum of (*o*-Fph)PPh.

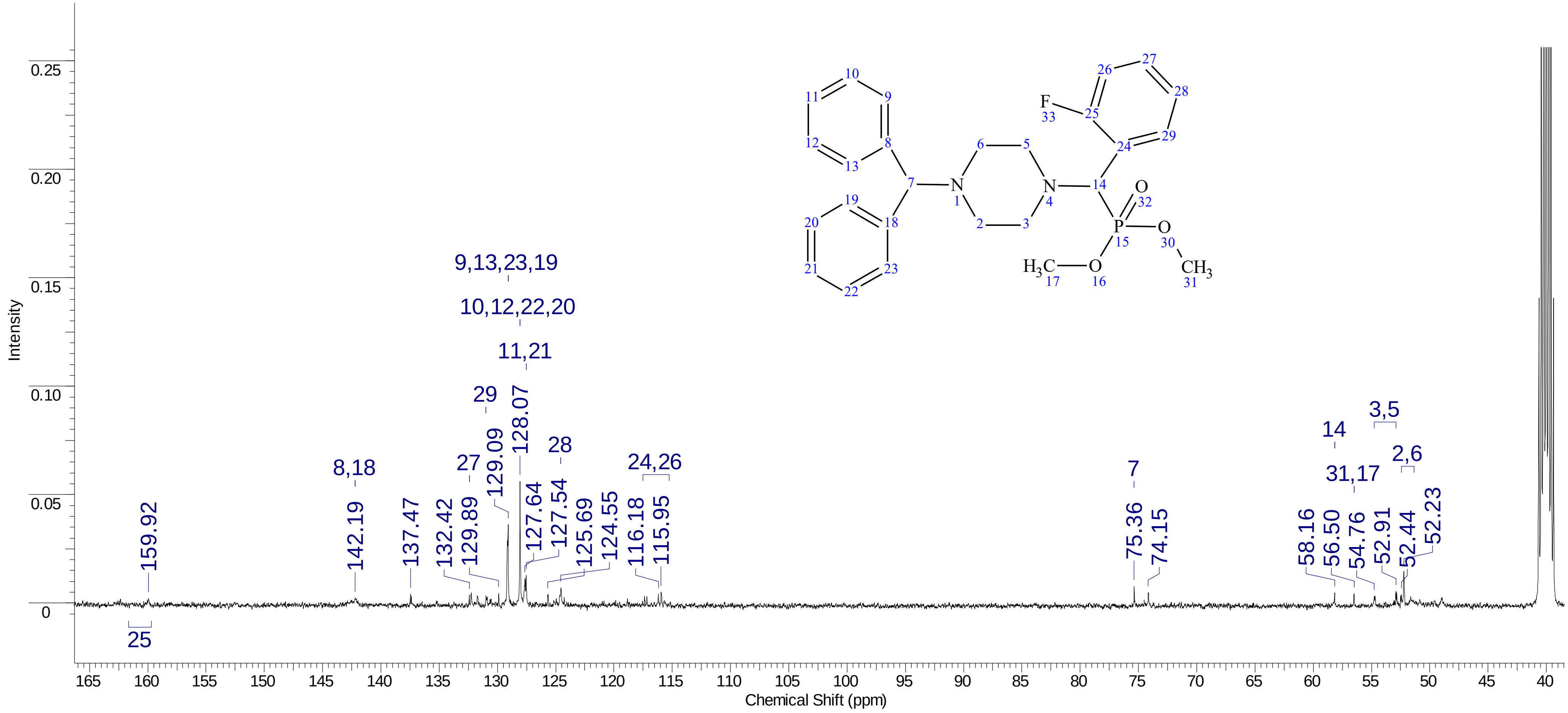

Figure S7. ^{13}C NMR (100.53 MHz, DMSO-d$_6$) spectrum of (*o*-Fph)PPh.

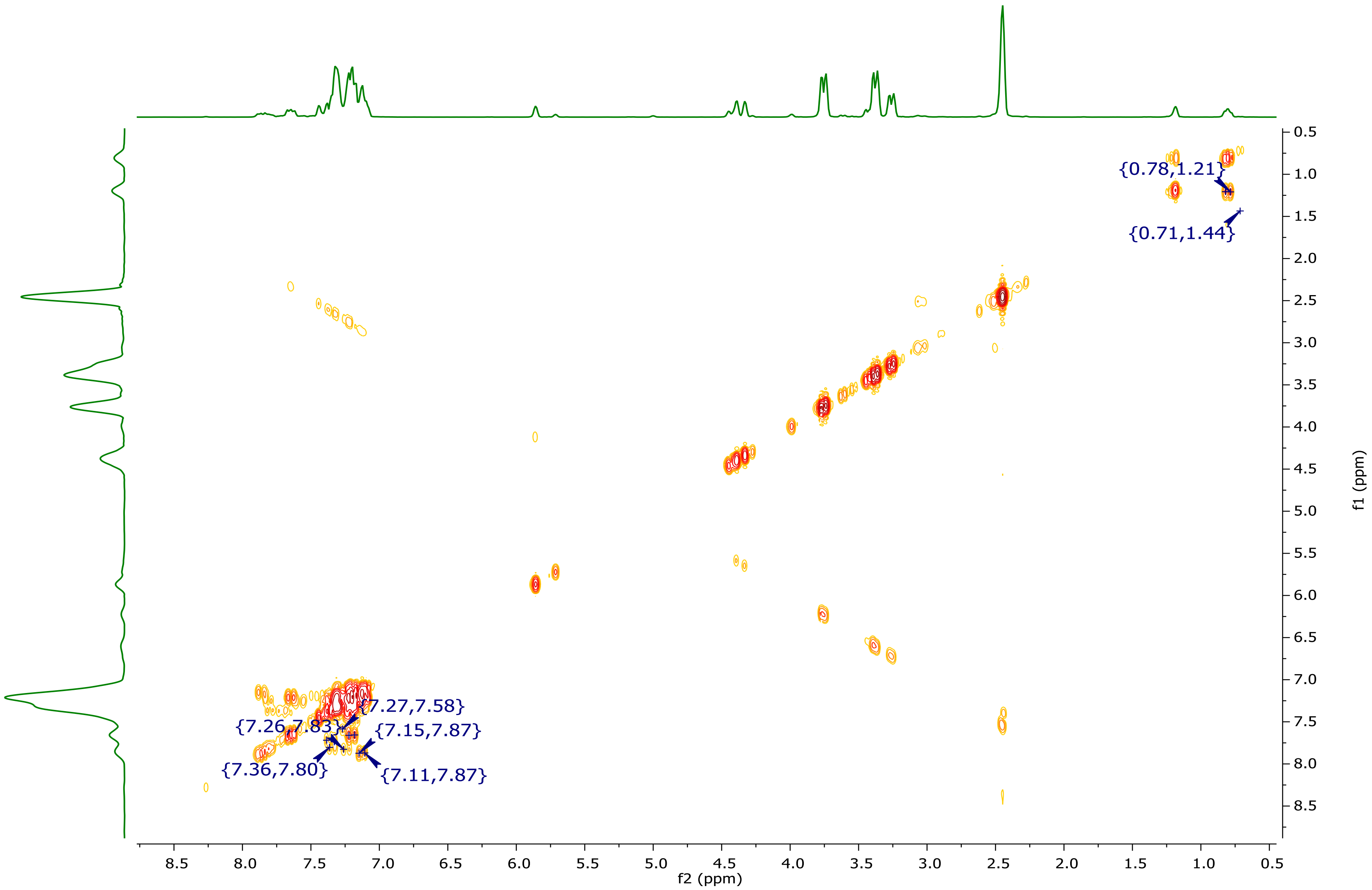

Figure S8. ^{1}H-^{1}H COSY spectrum of (*o*-Fph)PPh.

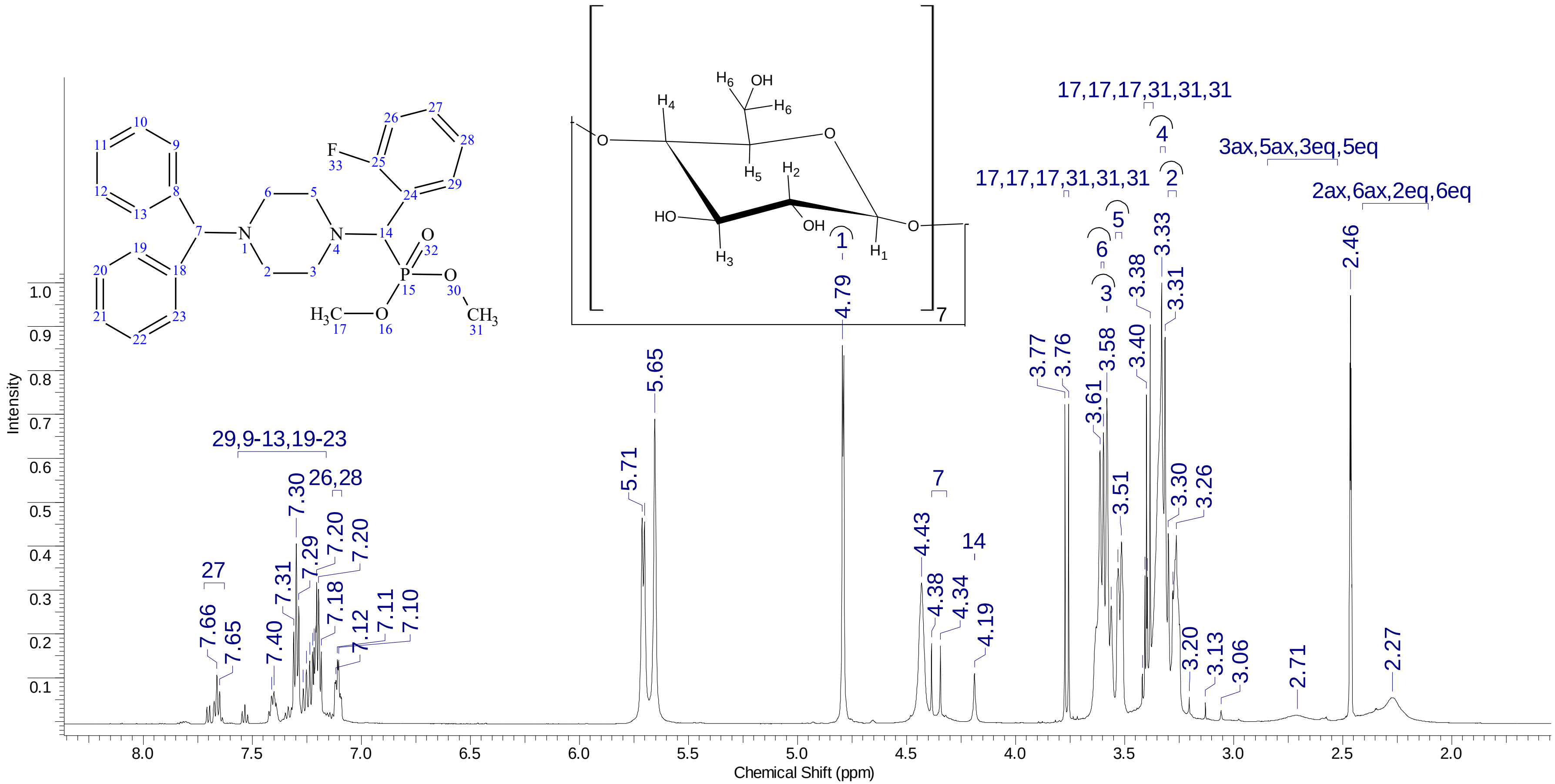

Figure S9. ^{1}H NMR (399.78 MHz, DMSO-d$_6$) spectrum of (***o*-Fph**)**PPhβCD**.

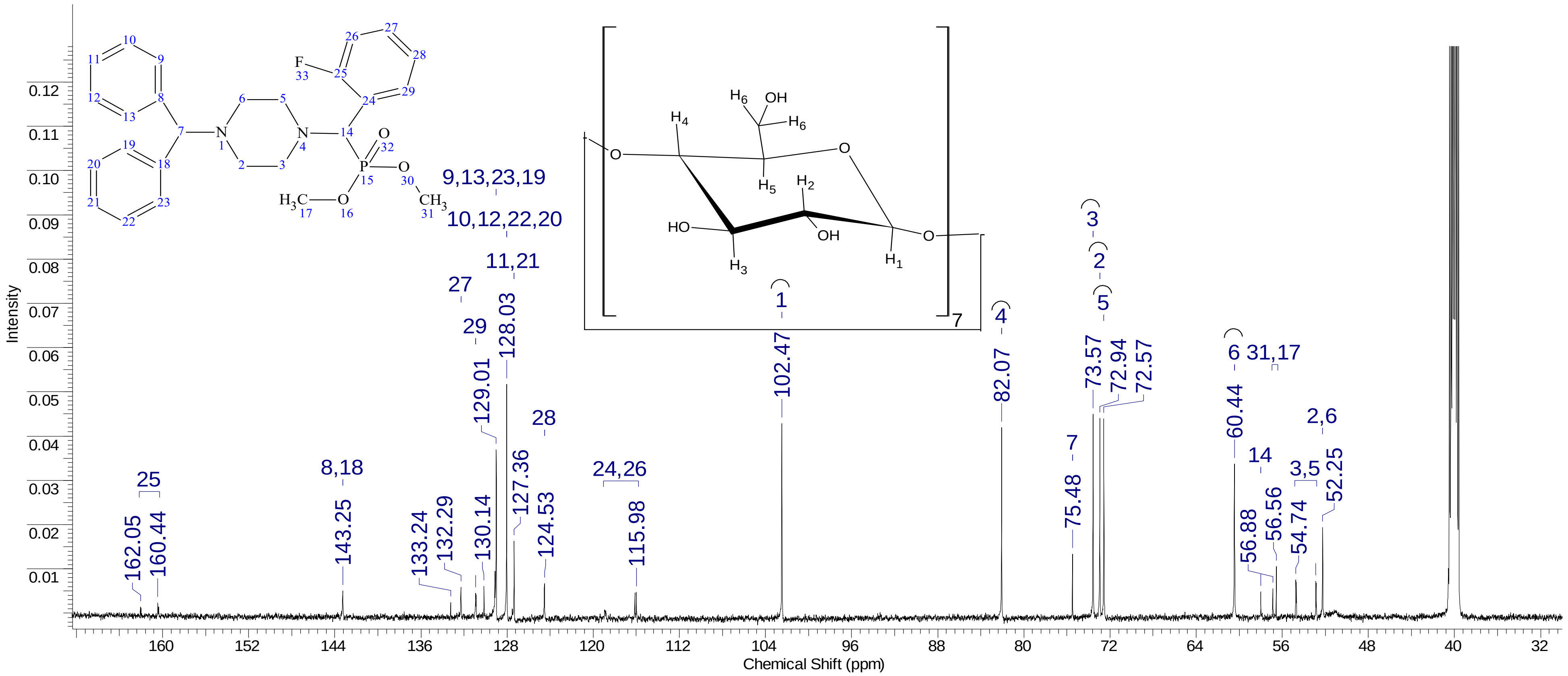

Figure S10. ^{13}C NMR (100.53 MHz, DMSO-d$_6$) spectrum of (***o*-Fph**)**PPhβCD**.

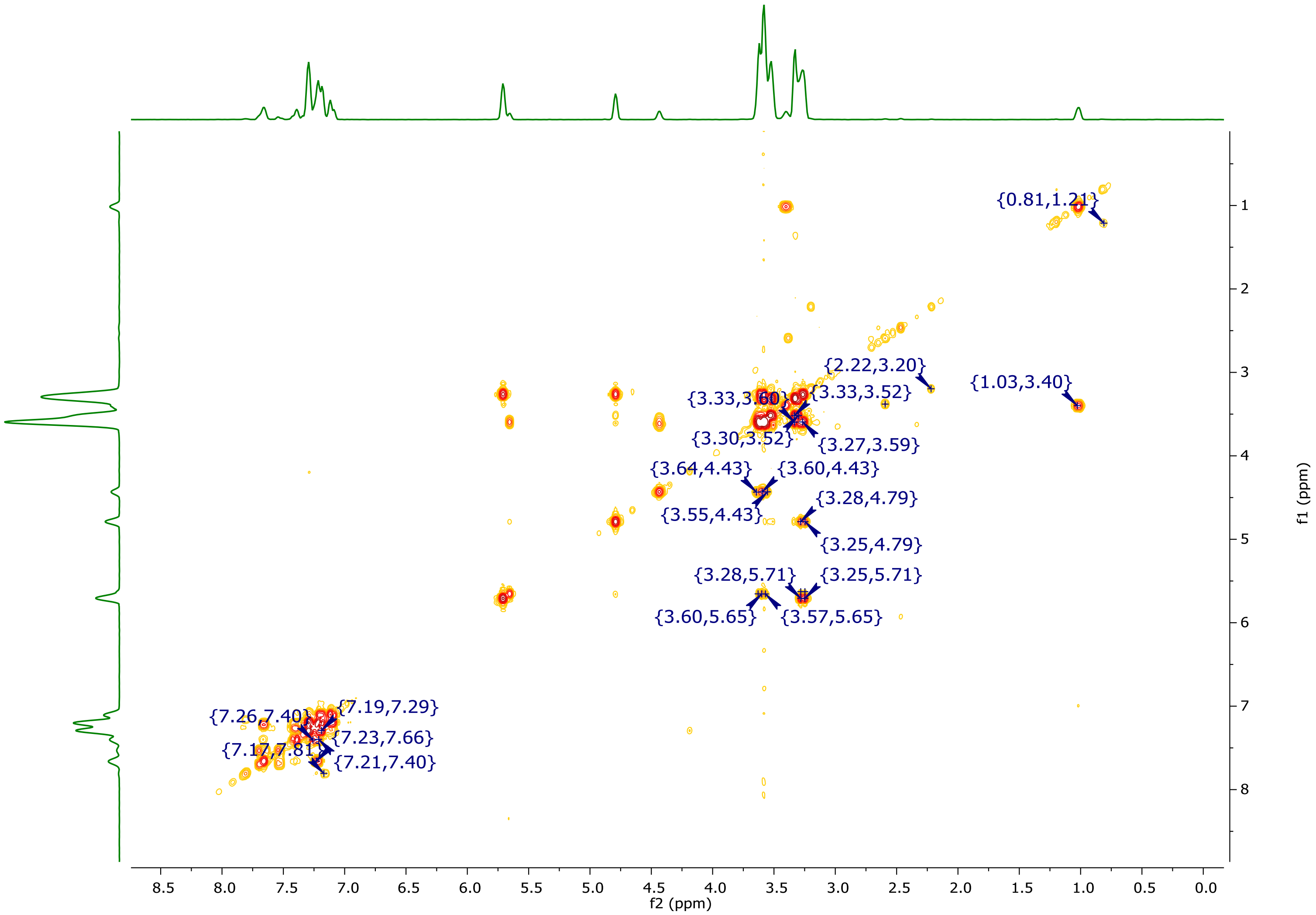

Figure S11. ^{1}H-^{1}H COSY spectrum of (***o*-Fph**)**PPhβCD.**

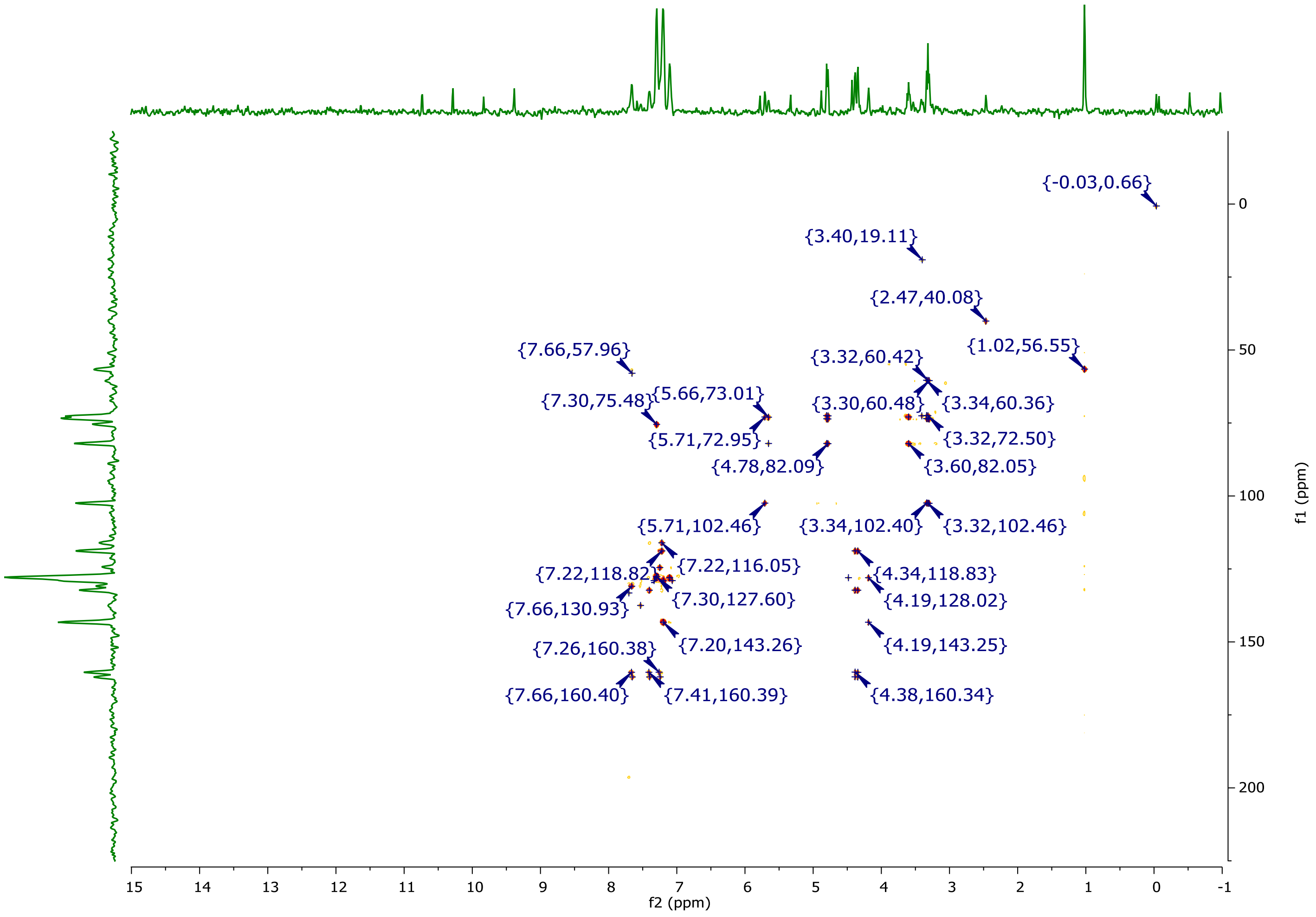

Figure S12. ^{1}H-^{13}C HMBC spectrum of (***o*-Fph**)**PPhβCD**.
